# Intraoperative morphometric study of distal femur in Brazilian patients undergoing total knee arthroplasty

**DOI:** 10.1371/journal.pone.0233715

**Published:** 2020-05-29

**Authors:** Fabrício Bolpato Loures, Rogério Franco de Araújo Góes, Eduardo Branco de Sousa, Naasson Cavanellas, João Maurício Barretto, Marcel Jun Sugawara Tamaoki, Rodrigo Sattaminni Pires e Albuquerque, Pedro José Labronici

**Affiliations:** 1 Orthopedic Surgery Department, Universidade do Estado do Rio de Janeiro (UERJ), Rio de Janeiro, RJ, Brazil; 2 Orthopedic Surgery Department, Hospital Santa Teresa (HST), Petrópolis, RJ, Brazil; 3 Orthopedic Surgery Department, Instituto Nacional de Traumatologia e Ortopedia (INTO), Rio de Janeiro, RJ, Brazil; 4 Orthopedic Surgery Department, Escola Paulista de Ortopedia, Universidade Federal de São Paulo (UNIFESP), São Paulo, SP, Brazil; 5 Orthopedic Surgery Department, Universidade Federal Fluminense (UFF), Niterói, RJ, Brazil; Monash University, AUSTRALIA

## Abstract

**Background:**

Total knee arthroplasty (TKA) is the treatment option for patients with severe osteoarthritis (OA) of the knee whose symptoms are refractory to conservative management. Unfortunately, the level of patient dissatisfaction is high, reaching up to 25%. The reasons for this dissatisfaction are multifactorial, but bone-implant mismatch significantly increases the chance of pain and functional limitation. Sex-specific prosthesis designs have been developed to overcome this issue, but their use is still controversial. The primary objective of this study was to evaluate possible sex differences in the shape of the distal femur in patients with osteoarthritis. Secondary objectives were to investigate interpersonal variability of the distal femur and to determine the number of femoral implant sizes required to meet shape variations.

**Methods and findings:**

A cross-sectional observational study prospectively compared 294 knees of 293 patients with osteoarthritis according to sex (201 female/93 male). Six intraoperative measurements were performed on the distal femur (height and width of both lateral and medial condyles, total medial-lateral width of the femur, and intercondylar distance). Sex differences and interpersonal variability were analyzed by multiple linear regressions. Measurements were also correlated with patient height. An optimization analysis was used to estimate the number of femoral implant sizes required. There were significant sex differences in the distal femur, where men had higher values than women in all measurements. Great interpersonal variability was found. The height of the lateral condyle was correlated with patient height, but the correlation was not strong. Twenty-five femoral implant sizes were required to meet the shape variations in our sample.

**Conclusions:**

The shape of the distal femur in patients with osteoarthritis shows great interpersonal variability, with men showing significantly higher values than women. A total of 25 different implant sizes would be necessary to adequately meet the variations observed in our study population.

## Introduction

Total knee arthroplasty (TKA) is the treatment option for patients with severe osteoarthritis (OA) of the knee whose symptoms are refractory to conservative management [1]. This intervention has proven effective in the treatment of knee OA by relieving pain, restoring limb alignment, and improving mobility [2]. Despite the success of this procedure, the level of patient dissatisfaction is still high, ranging from 11 to 25% [3]. Nearly 40% of patients believe their expectations have not been fully met by their TKA [4]. A perfect fit between the implant and the resected bone is essential to mimic the function of the knee. Gaps at the bone-implant interface increase the complexity of the procedure and the risk of complications, which may reduce the durability of TKA [5]. The number of designs and sizes of knee prostheses available for TKA has increased significantly in recent decades [5], but an undesirable bone-implant incompatibility still occurs in 28 to 68% of cases [6, 7].

The incidence of OA is 2–3 times higher in women than in men [8]. There is an open question as to whether the anatomy really differs between men and women and whether this difference could explain a greater risk of dissatisfaction among women, since nearly all TKA prostheses have been designed based on the anthropometric features of male patients [9, 10]. The literature is controversial in this regard, mainly due to the lack of standardization of study methods.

The primary objective of this study was to evaluate possible sex differences in the shape of the distal femur in patients with OA. Secondary objectives were to investigate interpersonal variability of the distal femur and to determine the number of femoral implant sizes required to meet shape variations.

## Materials and methods

### Patients

This comparative cross-sectional observational study was approved by the research ethics committee of the National Institute of Trauma and Orthopedics (number 1.426.956). All procedures were performed in accordance with the ethical standards of the institutional research committee and with the 1964 Helsinki declaration and its later amendments or comparable ethical standards. We recruited patients who underwent TKA at the National Institute of Trauma and Orthopedics and at Santa Teresa Hospital, both hospitals are located in the state of Rio de Janeiro, Brazil. All patients were born in Brazil, lived in the state of Rio de Janeiro, and belonged to different socioeconomic strata. Patients were operated on through the Brazilian public health care system, which provides comprehensive care for any citizen without copayments or patient charges. The Brazilian population has a mixed biological origin, with varying degrees of European, African, and Amerindian contribution. Therefore, the skin color phenotype has a weak association with ancestry.

Written informed consent was obtained from all individual participants prior to their inclusion in the study. A total of 294 knees (203 female/91 male) of 293 patients undergoing TKA between August 2012 and December 2016 were included in the study and prospectively evaluated according to sex (male/female). The mean patient age was 69.4±7.5 years for women and 68.3±7.9 years for men. The mean patient height was 1.57±0.10 m for women and 1.71±0.10 m for men. Patients were excluded if they had a history of fracture or previous knee surgery, if they had bone loss requiring grafting, or if the knee had a varus or valgus deformity greater than 15º. Patients were interviewed and examined according to the protocol for preoperative evaluation. With the patient wearing only underwear and an apron, weight was measured on a Welmy® W300 digital scale, with a capacity of up to 300 kg and a precision of 50 g, and height was measured using a 200-cm anthropometer to the nearest 0.5 cm. The anatomic axis of the limb to be operated on was measured on weight-bearing panoramic radiographs. The protocol was attached to the patient's chart and sent to the surgical center in order to be subsequently completed with data from intraoperative measurements. This protocol is registered at protocols.io [dx.doi.org/10.17504/protocols.io.behxjb7n]

### Intraoperative measurements

**Fig 1:**
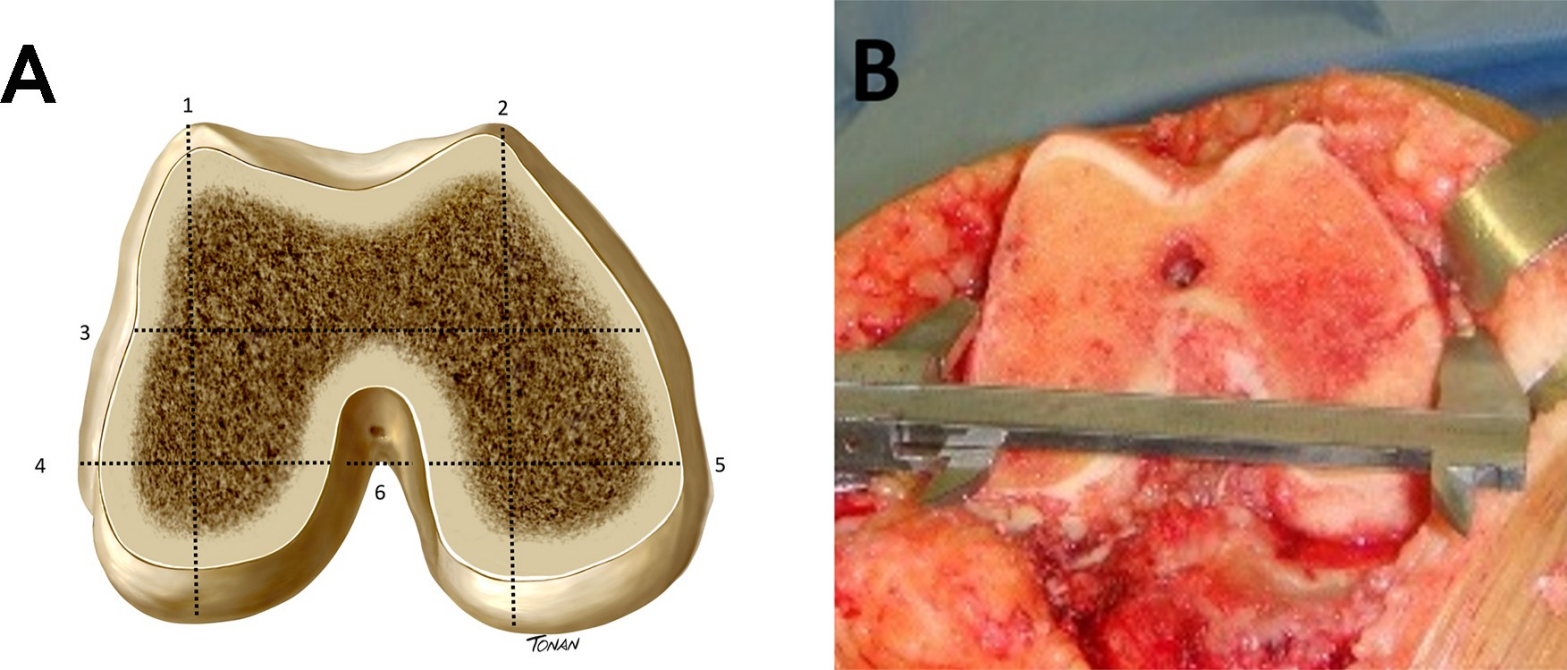
**A)** Schematic representation of intraoperative measurements in the left distal femur: 1) height of the lateral condyle; 2) height of the medial condyle; 3) total width of the femur; 4) width of the lateral condyle; 5) width of the medial condyle; 6) intercondylar distance [7]. **B)** Intraoperative measurement of distal femur (total width of the femur -TWF).

Six metal calipers were acquired and sent to the Institute of Metrology, Quality and Technology (INMETRO), which confirmed the precision of the instruments. During the surgical procedure, the primary surgeon (FBL, first author) made six femoral measurements (height and width of both lateral and medial condyles, total medial-lateral width of the femur, and intercondylar distance), as described by Loures et al. [6] (Fig 1). All measurements were recorded in millimeters (mm) and made in duplicate, with the second measurement being made by one of the coauthors (RFAG). The average of the two measurements was used for analysis, thus minimizing possible distortions. We did not take the changes in the anatomic axis into account in the analysis, but we excluded from the sample all patients with moderate to severe deformities (angular deviation greater than 15º); therefore, knees with important bone defects were not included.

Before the measurements, a distal femoral cut was made and all osteophytes were removed. This maneuver allowed for the identification of the real dimensions of the bone surfaces that would be in direct contact with the implant, as described by Chin et al. [11]. In all cases, the distal femoral cut was made using an intramedullary guide, with the angle of the distal resection set at 6° valgus and 9 mm from the distal edge of the femur. The medial-lateral width was measured as the distance between the medial and lateral cortices at the level of the surgical transepicondylar axis. The width of the femoral condyles was measured at 8 mm from the posterior articular surface of the lateral condyle and at 10 mm from the posterior articular surface of the medial condyle, simulating the external rotation of the femoral implant. Anterior-posterior measurements were performed before femoral cutting, perpendicular to the transepicondylar axis. The relative index (RI) between total width and height of the lateral condyle was also assessed, as described by Hitt et al. [12].

### Statistical analysis

Quantitative data were expressed as mean and standard deviation (SD), median and range, or coefficient of variation (CV) and SD, while categorical data were expressed as frequency (n) and percentage (%). In the inferential analysis of quantitative variables, the Kolmogorov-Smirnov and Shapiro-Wilk tests were used to assess the normality of data distribution. Data distribution was considered normal only if both tests indicated normality. Normally distributed data were analyzed using Student’s *t* test. Levene’s test was used to test for homogeneity of variance. When data were not normally distributed, two independent groups were compared using the nonparametric Mann-Whitney test. The binomial test was used to compare two complementary proportions. Correlation analysis was performed to investigate the association between two quantitative variables. Pearson’s correlation coefficient was used in cases of normal distribution, while Spearman’s correlation coefficient was used for confirmation in cases of non-Gaussian distribution. The correlation was considered significant at *P *< 0.05. A multiple linear regression model was used to examine the relationship between total width of the femur and height of the lateral condyle. The level of significance was set at 5% (*P *< 0.05) for all analyses. Data analysis was performed using SPSS, version 22.0.

An optimization analysis was used to estimate the number of implant sizes required to adequately meet the variations in the shape of distal femurs in the sample, respecting the maximum overhang or underhang of 3 mm, as described by Mahoney et al. [7]. An optimization analysis aims to find the optimum value for one or more target variables, given certain constraints. The variables are changed repeatedly, subject to the specified constraints, until the best value for the target variable is obtained. To this end, R software, version 3.5.0, was used to define, through computational analysis, the number of implant sizes required to meet the shape variations in our sample.

## Results

A total of 294 knees (203 female/91 male) were included in the study. The female-to-male ratio was 2.23: 1. The male and female groups did not differ in age (*P * =  0.293). The age and anthropometric features of the sample are shown in Table 1.

**Table 1:** Characteristics of the sample.

Variable	Females N = 203		Males N = 91		*P*-value
	Mean ± SD	Median	Mean ± SD	Median	
Age (years)	69.4±7.5	70	68.3±7.9	68	0.293*
Weight (kg)	75.1±13.6	75	84.6±14.8	82	<0.001**
Height (m)	1.57±0.10	1.57	1.71±0.10	1.70	<0.001**
BMI (kg/m^2^)	30.4±5.3	30.1	29.0±4.0	28.7	0.017**

BMI, body mass index.

* Mann-Whitney test

** Student’s *t* test.

In all measurements, male knees had higher mean values than female knees (*P* < 0.001). The ratio between the measurements of total width of the femur and height of the lateral condyle (RI) was lower in female knees (115%) than in male knees (116%), although there was no statistical difference (*P* = 0.183). All measurements were found to be not normally distributed and were analyzed by nonparametric tests. Femoral measurement results according to sex are shown in Table 2.

**Table 2:** Femoral measurements according to sex.

Variable	Females N = 203		Males N = 91		*P*-value
	Mean ± SD	Median	Mean ± SD	Median	
TWF	70.2±5.4	70	79.9±5.7	80	<0.001*
WLC	29.1±4.6	29	33.0±4.2	33	<0.001*
WMC	29.0±4.8	28	33.7±6.9	33	<0.001*
Intercondylar distance	15.1±3.5	15	16.8±3.9	17	<0.001*
HLC	61.6±5.2	62	68.4±5.6	69	<0.001*
HMC	60.7±6.2	61	68.6±5.7	68	<0.001*
*Ratio* F**	1.15±0.1	1.13	1.16±0.1	1.14	0.183*

Values in millimeters.

TWF, total width of the femur; WLC, width of the lateral condyle; WMC, width of the medial condyle; HLC, height of the lateral condyle; HMC, height of the medial condyle.

* Mann-Whitney test

** Ratio of the total width of the femur to the height of the lateral condyle.

**Fig 2:**
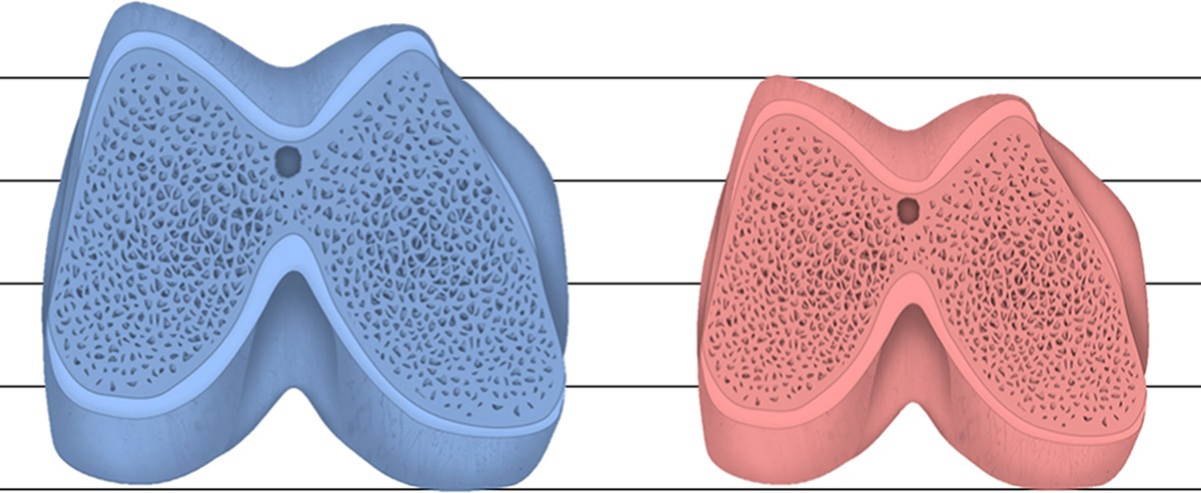
Proportional size difference between male and female distal femurs.

Based on relative measurements, the mean total width of the femur was 13.8% greater in men than in women. The mean height of the lateral condyle and the medial area of the distal femoral epiphysis were, respectively, 11.9% and 27.3% greater in men than in women. Fig 2 shows the proportional size difference between male and female femurs.

**Fig 3:**
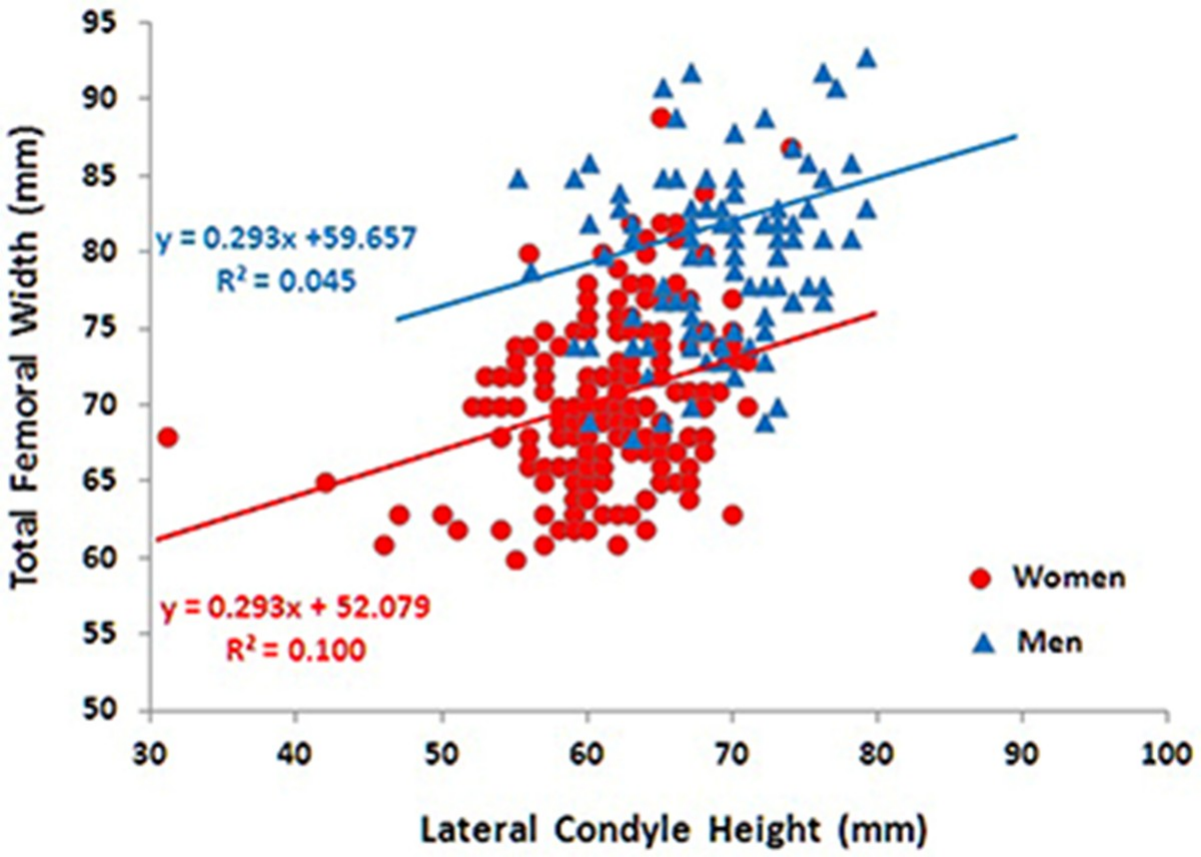
Multiple linear regression analysis between male and female distal femurs.

To determine the variation in distal femur shape, the relationship between the total width of the femur and the height of the lateral condyle was evaluated based on the comparison of multiple regression lines. Multiple linear regression analysis, through the parallel test, showed no significant difference in the RI between men and women with OA (*P  =  *0.329) (Fig 3).

Although the CV for the RI was considered statistically low both in the male group (CV  =  0.10) and in the female group (CV  =  0.11), the RI varied widely in both sexes. A direct positive relationship was found between TWF and HCL measurements (*P* = 0.000). Although a moderate correlation was observed for the total sample (r = 0.521), this correlation was weak for females (r = 0.302) and very weak for males (r = 0.205), demonstrating the large number of possible combinations between the two measurements (Table 3).

**Table 3:** Correlation between TWF and HLC.

	Sex	Spearman’s coefficient	Pearson’s coefficient
Overall	Correlation coefficient	0.530	0.521
	*P*-value	0.000	0.000
Females N = 203	Correlation coefficient	0.318	0.302
	*P*-value	0.000	0.000
Males N = 91	Correlation coefficient	0.214	0.205
	*P*-value	0.042	0.042

TWF, total width of the femur; HLC, height of the lateral condyle

**Fig 4:**
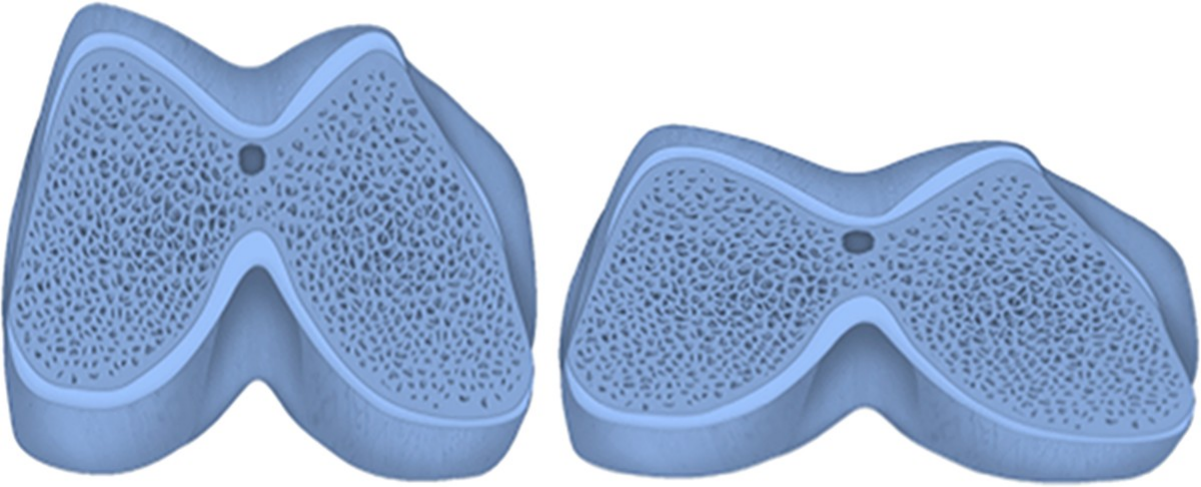
Variation in male distal femurs: Narrowest femur (RI  =  0.96) and widest femur (RI  =  1.54). RI, relative index.

**Fig 5:**
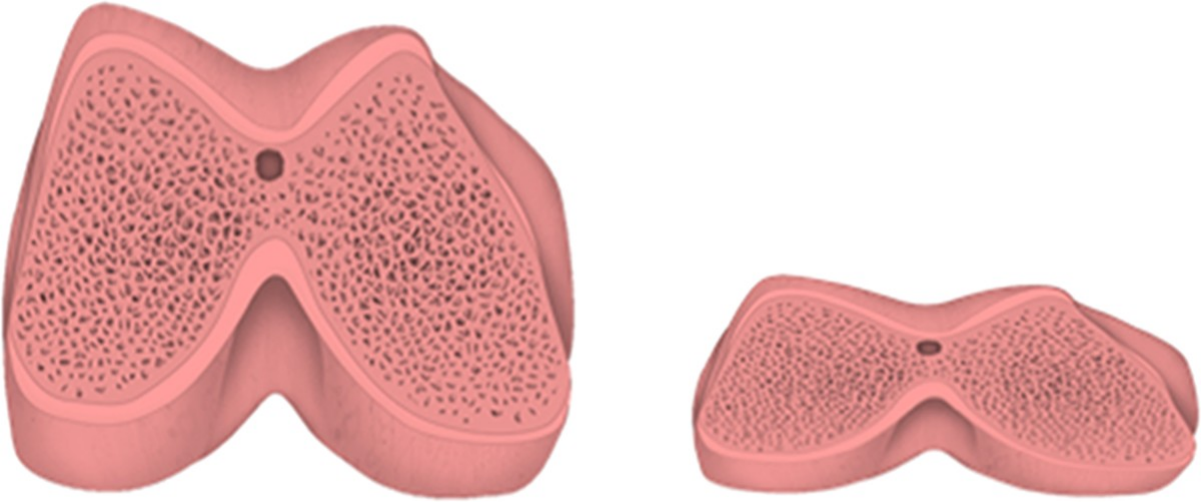
Variation in female distal femurs: Narrowest femur (RI  =  0.90) and widest femur (RI  =  2.19). RI, relative index.

The combination of these measurements results in the great variation at the distal end of the femur between individuals. Fig 4 shows the proportional difference between the narrowest (RI  =  0.96) and widest (RI  =  1.54) male femur. Fig 5 shows the proportional difference between the narrowest (RI  =  0.90) and widest (RI = 2.19) female femur.

The mean height of men was higher than that of women (*P *< 0.001). The height of the lateral condyle was correlated with patient height (*P *< 0.05), but the correlation was not strong (r* *< 0.7). This association was not confirmed for total width of the femur or RI.

**Fig 6:**
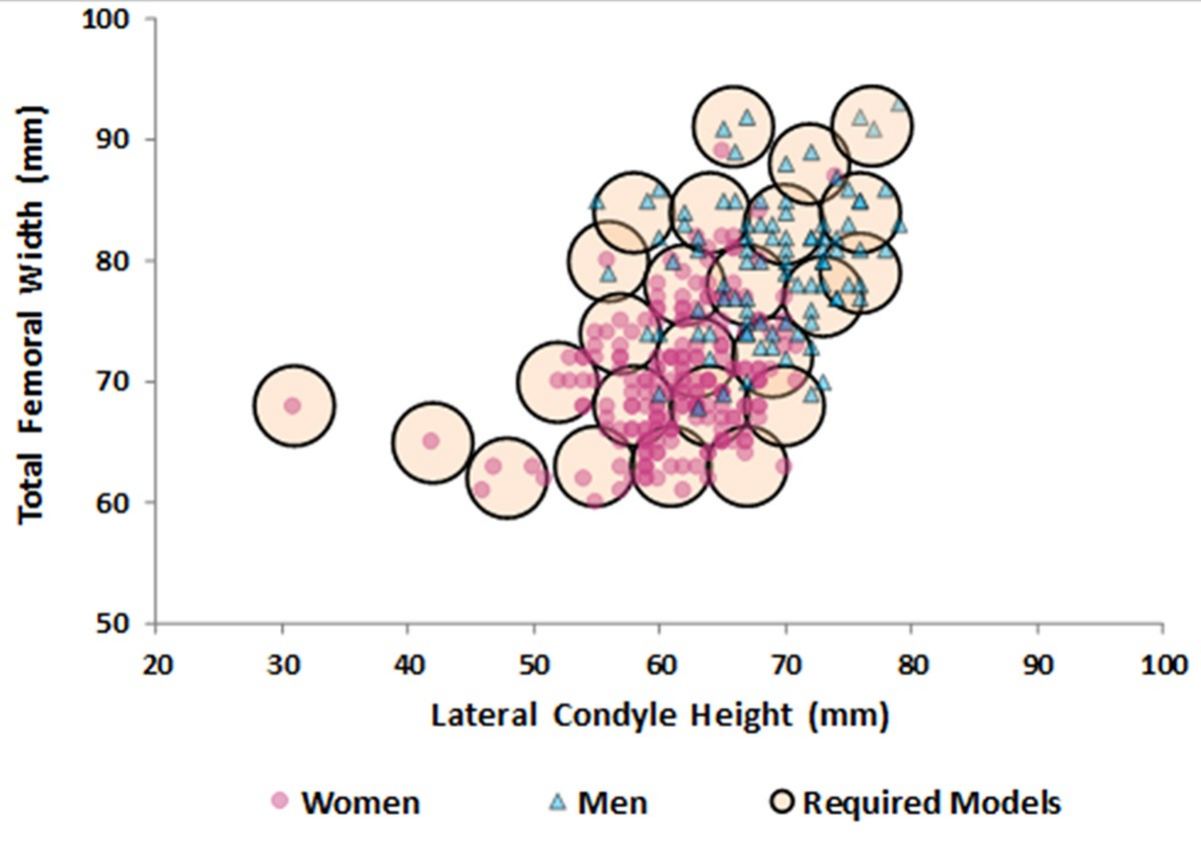
Estimated number of femoral implant sizes required to meet shape variations.

Considering an overhang or underhang of up to 3 mm, the optimization analysis revealed that 25 implant sizes would be necessary to adequately meet the shape variations in our study population. This estimation is illustrated in Fig 6.

## Discussion

Many factors can influence the outcome of TKA, such as preoperative knee range of motion, surgical technique, accuracy of implant placement, rehabilitation process, and prosthesis model [13–16]. Since the early 1980s, mismatched implant sizes have been identified as a causative factor of pain and functional limitation after TKA [17]. Classically, femoral implants are chosen based on the anterior-posterior length of the distal femur to provide a normal gait and a stable joint throughout the range of motion [18]. However, this choice may lead to inadequate coverage of the distal end of the femur in the medial-lateral direction. In cases of femoral component underhang, radiolucent lines may appear on the bone-implant interface, with increased postoperative bleeding, which can lead to hematoma formation and impaired wound healing [19]. Exposure of cancellous bone to polyethylene wear debris can cause radiolucent lines to appear around the prosthesis, leading to early implant loosening. When there is femoral component overhang, proper ligament balance may be compromised, leading to postoperative pain and decreased range of motion [7]. Therefore, the shape of the prosthesis plays a crucial role in clinical outcome.

The reasons for patient dissatisfaction after TKA are multifactorial [13], but it is clear that the correct fit between implant and resected bone is essential for good clinical outcome and durable joint replacements [20]. The results of the present study demonstrated great anatomic variability in the shape of the distal femur, which jeopardizes proper fitting of the implant to the resected bone. This finding supports the high rate of incompatibility described by Loures et al. [6], who compared the dimensions of implants with those of resected bones and found incompatibilities greater than 3 mm in 28% of cases. Bourne et al. [3] evaluated 1703 patients undergoing TKA and reported a 19% rate of dissatisfaction with the outcome, with no sex differences. The authors compared their data with the literature and found that satisfaction levels over the past 20 years were not different than that reported in their study, suggesting that advances in implant design have been insufficient to improve clinical outcomes.

Conley et al. [17], based on anatomic studies, published an article in 2007 presenting a sex-specific prosthesis tailored to meet the specific needs of female patients (*Gender Solutions*®; Zimmer Inc, Warsaw, IN). Among other modifications, this implant has a narrower femur to allow better fitting to the resected bone. Colleoni et al. [21] evaluated 30 women undergoing TKA, 15 with conventional implants and 15 with sex-specific implants. After 5 years of follow-up, functional results were similar in the two groups, demonstrating that the anatomical advantages proposed by *Gender Solutions*® do not translate into functional or clinical results [13]. We found that male knees were significantly bigger than female knees, which is consistent with the literature [22–24]. Yang et al. [25], based on simulated distal femur cuts in 130 osteoarthritic knees, also found significant differences between men and women. However, the proposed adjustments in the shape of sex-specific implants seem to be insufficient to meet the wide existing variations, even those within the same group.

The combination between medial-lateral and anteroposterior (RI) width measurements allows us to predict the shape of the distal femur [26], indicating a rectangular/trapezoidal variability [24]. A wide interpersonal variation was observed in the present study, with the RI showing a 1.6-fold variation in men and a 2.43-fold variation in women. Several patients with the same height of the lateral condyle, which is the parameter of choice for femoral implant, showed different measurements of total width of the femur. However, the implants had a constant RI, which forces surgeons to perform adaptive measurements intraoperatively [18]. These results are consistent with Hitt et al. [12], who evaluated 337 knees during TKA and concluded that implants need changes to better match the shape of resected bones. Among patients undergoing bilateral TKA, 31% used femoral prostheses of different sizes in each knee, confirming the wide variation in knee shape even within the same individual. This great variation often results from mismatch between implant geometry and knee morphology, which could lead to pain and stiffness after TKA [6;27].

We investigated a possible association between knee morphometry and factors such as sex, age, stature, and stage of OA, but none of these factors could reliably predict the shape of the distal femur. These findings are in accordance with Bellemans et al. [28], who analyzed the shape of the knee in 1000 patients undergoing TKA, classifying patients according to body type as endomorph, mesomorph, or ectomorph, and found a large variability between narrow and wide knees, even within the same sex, concluding that multiple factors are associated with this large variability. Our study indicates a direct relationship between patient height and lateral condyle height (*P *< 0.05). These results are in accordance with Piriou et al. [29], who measured 376 knees with a computerized surgical navigation system during TKA and found a direct relationship between distal femoral epiphysis dimensions and femur length, which can be interpreted as an individual’s height. However, this relationship was weak (r* *< 0.7) to be considered predictive of femoral shape.

Our sample came from a multiracial country, where the phenotype is a weak predictor of genetic heritage, and we considered it an advantage that strengthens our results [30]. Some studies have attempted to demonstrate biological origin as an indicator of knee shape [10, 31], but it is not clear whether this difference is clinically important, and with the continuing process of globalization, this variation tends to be minimized. Research has been conducted to better understand the anatomy of the knee in an attempt to recreate its biomechanics with minimal constraint to mobility and implant wear. Many factors can influence the shape of the distal femur, such as stature, biological origin, morphotype, age, sex, and stage of OA, so it seems a difficult task to consider all these variables simultaneously. Thus, we believe that, in patients with OA who are candidates for TKA, the distal femur should be seen as an individual feature. Improvements in the design of TKA components are critical to obtain the ideal fit that will allow the best function [32].

To adequately meet the shape variations in our sample, 25 different femoral implant sizes would be necessary. To this end, in the current production model, manufacturing, logistics and stocking costs would increase significantly, which could have a great impact on the health care system. Recent health care changes have required cost reduction and increased effectiveness [33]. New technologies are bringing greater precision to TKA and may help to improve this relationship. Although with short-term results, Culler et al. [34] compared 126 customized implants with 122 standard off-the-shelf implants and found better rehabilitation results for the former. Customized implants were more expensive, but the final hospital expenditure was similar (*P* = 0.913), justified by a lower rate of adverse events. Studies with longer follow-up must be conducted to ensure that a more precise matching of the implant to the resected bone surface is really reflected in better clinical results and fewer revisions.

This study has some limitations. The comparisons were based on metric measurements between distinct points on the femur, but this is not a true three-dimensional analysis [32]. Besides that, intraoperative measurements may vary slightly due to the positioning of the caliper. Theses metric methods suffer from analysis bias related to inter- and intraobserver errors and standardization challenges [35]. However, we believe that surgeons highly experienced in arthroplasty are able to accurately locate the anatomic landmarks.

That measurements were performed *in vivo*, after preparing the bone surface to receive the implant, avoids the distortions that may be introduced by the imaging device. All patients analyzed had a diagnosis of knee OA and, therefore, had the typical anatomic changes of the joint submitted to arthroplasty, as described by Cheng et al. [32], Matsuda et al. [36], and Puthumanapully et al. [37]. The mean age of the sample was 70 years, comprising only candidates for TKA. This was an important characteristic of the study, as the anatomy of the femur is influenced by age, as demonstrated by Pujol et al. [38,39], Cavaignac et al. [40], and Li et al. [41]. The significant number of patients included enhances the power of inference in the general population.

In conclusion, the present study found that the shape of the distal femur in patients with OA shows great interpersonal variability, with men showing significantly higher measurement values than women. These variations should be taken into account when designing TKA components intended to better match the natural geometry of the femur in men and women. The results suggest that a total of 25 different implant sizes would be necessary to adequately meet the shape variations observed in our study population.

## Supporting information

S1 Checklist STROBE statement—checklist of items that should be included in reports of observational studies. (DOCX)
