# Supplementary material for: Intraoperative morphometric study of distal femur in Brazilian patients undergoing total knee arthroplasty
Source: PLoS One. 2020 May 29;15(5):e0233715. doi: 10.1371/journal.pone.0233715 (PMC7259597; doi:10.1371/journal.pone.0233715)
Supplement: S1 Checklist — (DOCX) [file pone.0233715.s001.docx]

STROBE Statement—checklist of items that should be included in reports of observational studies

|  | Item No. | Recommendation | Page  No. | Relevant text from manuscript |
| --- | --- | --- | --- | --- |
| **Title and abstract** | 1 | (*a*) Indicate the study’s design with a commonly used term in the title or the abstract | P.2, lines 30-31 | A cross-sectional observational study prospectively compared 294 knees of 293 patients with osteoarthritis according to gender (201 female/93 male). |
|  |  | (*b*) Provide in the abstract an informative and balanced summary of what was done and what was found | P.2, lines 32-41 | Six intraoperative measurements were performed on the distal femur (height and width of both lateral and medial condyles, total medial-lateral width of the femur, and intercondylar distance). Gender differences and interpersonal variability were analyzed by multiple linear regressions. Measurements were also correlated with patient height. An optimization analysis was used to estimate the number of femoral implant sizes required. There were no significant gender differences in the shape of the distal femur, but men had higher absolute values than women. Great interpersonal variability was found. The height of the lateral condyle was correlated with patient height, but the correlation was not strong. Twenty-five femoral implant sizes were required to meet the shape variations in our sample. |
| Introduction | | | |  |
| Background/rationale | 2 | Explain the scientific background and rationale for the investigation being reported | P. 4, lines 61-65 | There is an open question as to whether the anatomy really differs between men and women and whether this difference could explain a greater risk of dissatisfaction among women, since nearly all TKA prostheses have been designed based on the anthropometric features of male patients [9, 10]. The literature is controversial in this regard, mainly due to the lack of standardization of study methods. |
| Objectives | 3 | State specific objectives, including any prespecified hypotheses | P. 4, lines 66-69 | The primary objective of this study was to evaluate possible gender differences in the shape of the distal femur. Secondary objectives were to investigate interpersonal variability of the distal femur and to determine the number of femoral implant sizes required to meet shape variations. |
| Methods | | | |  |
| Study design | 4 | Present key elements of study design early in the paper | P. 5, lines 74-75 | This comparative cross-sectional observational study was approved by the institutional research ethics committee. |
| Setting | 5 | Describe the setting, locations, and relevant dates, including periods of recruitment, exposure, follow-up, and data collection | P. 5, lines 79-81 | A total of 294 knees of 293 patients undergoing TKA between August 2012 and December 2016 were included in the study and prospectively evaluated according to gender (male/female). |
| Participants | 6 | (*a*) *Cohort study*—Give the eligibility criteria, and the sources and methods of selection of participants. Describe methods of follow-up  *Case-control study*—Give the eligibility criteria, and the sources and methods of case ascertainment and control selection. Give the rationale for the choice of cases and controls  *Cross-sectional study*—Give the eligibility criteria, and the sources and methods of selection of participants | P. 5, lines 79-83 | A total of 294 knees of 293 patients undergoing TKA between August 2012 and December 2016 were included in the study and prospectively evaluated according to gender (male/female). Patients were excluded if they had a history of fracture or previous knee surgery, if they had bone loss requiring grafting, or if the knee had a varus or valgus deformity greater than 15º. |
|  |  | (*b*) *Cohort study*—For matched studies, give matching criteria and number of exposed and unexposed  *Case-control study*—For matched studies, give matching criteria and the number of controls per case | N/A |  |
| Variables | 7 | Clearly define all outcomes, exposures, predictors, potential confounders, and effect modifiers. Give diagnostic criteria, if applicable | N/A |  |
| Data sources/ measurement | 8* | For each variable of interest, give sources of data and details of methods of assessment (measurement). Describe comparability of assessment methods if there is more than one group | P. 6, lines 94-98 | Six metal calipers were acquired and sent to the Institute of Metrology, Quality and Technology (INMETRO), which confirmed the precision of the instruments. During the surgical procedure, the primary surgeon made six femoral measurements (height and width of both lateral and medial condyles, total medial-lateral width of the femur, and intercondylar distance), as described by Loures et al. [7] (Fig 1). |
| Bias | 9 | Describe any efforts to address potential sources of bias | P. 6, lines 98-100 | All measurements were made in duplicate and recorded in millimeters (mm). The average of the two measurements was used for analysis. |
| Study size | 10 | Explain how the study size was arrived at | P. 7, lines 120-123 | Assuming a level of significance of 5% (α) and a power of 80% (1-β), a sample size of at least 192 patients, 128 women and 64 men, was required to detect a relatively moderate correlation (r > 0.30) between two variables (total width of the femur and height of the lateral condyle or medial-lateral width). |
| Quantitative variables | 11 | Explain how quantitative variables were handled in the analyses. If applicable, describe which groupings were chosen and why | P. 7, lines 126-128 | Quantitative data were expressed as mean and standard deviation (SD), median and range, or coefficient of variation (CV) and SD, while categorical data were expressed as frequency (n) and percentage (%). |
| Statistical methods | 12 | (*a*) Describe all statistical methods, including those used to control for confounding | P. 7-8, lines 128-144 | In the inferential analysis of quantitative variables, the Kolmogorov-Smirnov and Shapiro-Wilk tests were used to assess the normality of data distribution. Data distribution was considered normal only if both tests indicated normality. Normally distributed data were analyzed using Student’s *t* test. Levene’s test was used to test for homogeneity of variance. When data were not normally distributed, two independent groups were compared using the nonparametric Mann-Whitney test. The binomial test was used to compare two complementary proportions. Correlation analysis was performed to investigate the association between two quantitative variables. Pearson’s correlation coefficient was used in cases of normal distribution, while Spearman’s correlation coefficient was used in cases of non-Gaussian distribution. The correlation was considered significant at *P*< 0.05. A multiple linear regression model was used to examine the relationship between total width of the femur and height of the lateral condyle. The level of significance was set at 5% (*P*< 0.05) for all analyses. Data analysis was performed using SPSS, version 22.0.  An optimization analysis was used to estimate the number of implant sizes required to adequately meet the variations in the shape of distal femurs in the sample, respecting the maximum overhang or underhang of 3 mm. |
|  |  | (*b*) Describe any methods used to examine subgroups and interactions | N/A |  |
|  |  | (*c*) Explain how missing data were addressed | N/A |  |
|  |  | (*d*) *Cohort study*—If applicable, explain how loss to follow-up was addressed  *Case-control study*—If applicable, explain how matching of cases and controls was addressed  *Cross-sectional study*—If applicable, describe analytical methods taking account of sampling strategy | N/A |  |
|  |  | (*e*) Describe any sensitivity analyses | P 7, lines 140-141 | The level of significance was set at 5% (*P*< 0.05) for all analyses. |
| Results | | | | |
| Participants | 13* | (a) Report numbers of individuals at each stage of study—eg numbers potentially eligible, examined for eligibility, confirmed eligible, included in the study, completing follow-up, and analysed | P 8, lines 147-148 | A total of 294 knees (203 female/91 male) were included in the study. The female-to-male ratio was 2.23: 1. |
|  |  | (b) Give reasons for non-participation at each stage | N/A |  |
|  |  | (c) Consider use of a flow diagram | N/A |  |
| Descriptive data | 14* | (a) Give characteristics of study participants (eg demographic, clinical, social) and information on exposures and potential confounders | P. 8, line 151 table 1 | Table 1. Characteristics of the sample. |
|  |  | (b) Indicate number of participants with missing data for each variable of interest | N/A |  |
|  |  | (c) *Cohort study*—Summarise follow-up time (eg, average and total amount) | N/A |  |
| Outcome data | 15* | *Cohort study*—Report numbers of outcome events or summary measures over time | N/A |  |
|  |  | *Case-control study—*Report numbers in each exposure category, or summary measures of exposure | N/A |  |
|  |  | *Cross-sectional study—*Report numbers of outcome events or summary measures | P. 9, line 161 Table 2 | Table 2. Femoral measurements according to gender. |
| Main results | 16 | (*a*) Give unadjusted estimates and, if applicable, confounder-adjusted estimates and their precision (eg, 95% confidence interval). Make clear which confounders were adjusted for and why they were included | N/A |  |
|  |  | (*b*) Report category boundaries when continuous variables were categorized | N/A |  |
|  |  | (*c*) If relevant, consider translating estimates of relative risk into absolute risk for a meaningful time period | N/A |  |
| Other analyses | 17 | Report other analyses done—eg analyses of subgroups and interactions, and sensitivity analyses | 10, lines 184-190 | Although the CV for the RI was considered statistically low both in the male group (CV = 0.10) and in the female group (CV = 0.11), the RI varied widely in both genders. This indicates that there are differences in the shape of the distal end of the femur between individuals, regardless of gender. Fig 4 shows the proportional difference between the narrowest (RI = 0.96) and widest (RI = 1.54) male femur. Fig 5 shows the proportional difference between the narrowest (RI = 0.90) and widest (RI = 2.19) female femur. |
| Discussion | | | | |
| Key results | 18 | Summarise key results with reference to study objectives | P. 12 , lines 236-239 | However, the results from our multiple linear regression analysis showed that anatomic differences in femur shape between men and women are not significant. The RI, which can be interpreted as a predictor of distal femoral shape, confirmed this hypothesis. |
| Limitations | 19 | Discuss limitations of the study, taking into account sources of potential bias or imprecision. Discuss both direction and magnitude of any potential bias | P. 13-14, lines 271-278 | This study has some limitations. Intraoperative measurements may vary slightly due to the positioning of the caliper. However, we believe that surgeons highly experienced in arthroplasty are able to accurately locate the anatomic landmarks. Also, all measurements were made twice, and the average of the two measurements was used for analysis, thus minimizing possible distortions. We did not take the changes in the anatomic axis into account in the analysis. However, we excluded from the sample all patients with moderate to severe deformities (angular deviation greater than 15º); therefore, important bone deformities were not included in the sample |
| Interpretation | 20 | Give a cautious overall interpretation of results considering objectives, limitations, multiplicity of analyses, results from similar studies, and other relevant evidence | P. 13, lines 261-263 | Our study confirmed that there is a direct relationship between patient height and lateral condyle height (*P*< 0.05), but this relationship was too weak (r < 0.7) to be considered predictive of femoral shape. |
| Generalisability | 21 | Discuss the generalisability (external validity) of the study results | P. 14, lines 278-287 | Strengths of this study include the fact that measurements were performed *in vivo*, after preparing the bone surface to receive the implant, which avoids the distortions that may be introduced by the imaging device. All patients analyzed had a diagnosis of knee osteoarthritis and, therefore, had the typical anatomic changes of the joint submitted to arthroplasty, as described by Cheng et al. [29]. The significant number of patients included enhances the power of inference in the general population. |
| Other information | |  | | |
| Funding | 22 | Give the source of funding and the role of the funders for the present study and, if applicable, for the original study on which the present article is based | P. 14, line 289 | The authors have no financial relationships relevant to this article to disclose. |

*Give information separately for cases and controls in case-control studies and, if applicable, for exposed and unexposed groups in cohort and cross-sectional studies.

**Note:** An Explanation and Elaboration article discusses each checklist item and gives methodological background and published examples of transparent reporting. The STROBE checklist is best used in conjunction with this article (freely available on the Web sites of PLoS Medicine at http://www.plosmedicine.org/, Annals of Internal Medicine at http://www.annals.org/, and Epidemiology at http://www.epidem.com/). Information on the STROBE Initiative is available at www.strobe-statement.org.
